# Supplementary material for: A multi-task domain-adapted model to predict chemotherapy response from mutations in recurrently altered cancer genes
Source: iScience. 2025 Feb 11;28(3):111992. doi: 10.1016/j.isci.2025.111992 (PMC11952854; doi:10.1016/j.isci.2025.111992)
Supplement: Document S1. Figures S1–S5 and Tables S1–S23 [file mmc1.pdf]

iScience, Volume *28*

Supplemental information

A multi-task domain-adapted model to predict chemotherapy response from mutations in recurrently altered cancer genes

Aishwarya Jayagopal, Robert J. Walsh, Krishna Kumar Hariprasannan, Ragunathan Mariappan, Debabrata Mahapatra, Patrick William Jaynes, Diana Lim, David Shao Peng Tan, Tuan Zea Tan, Jason J. Pitt, Anand D. Jeyasekharan, and Vaibhav Rajan

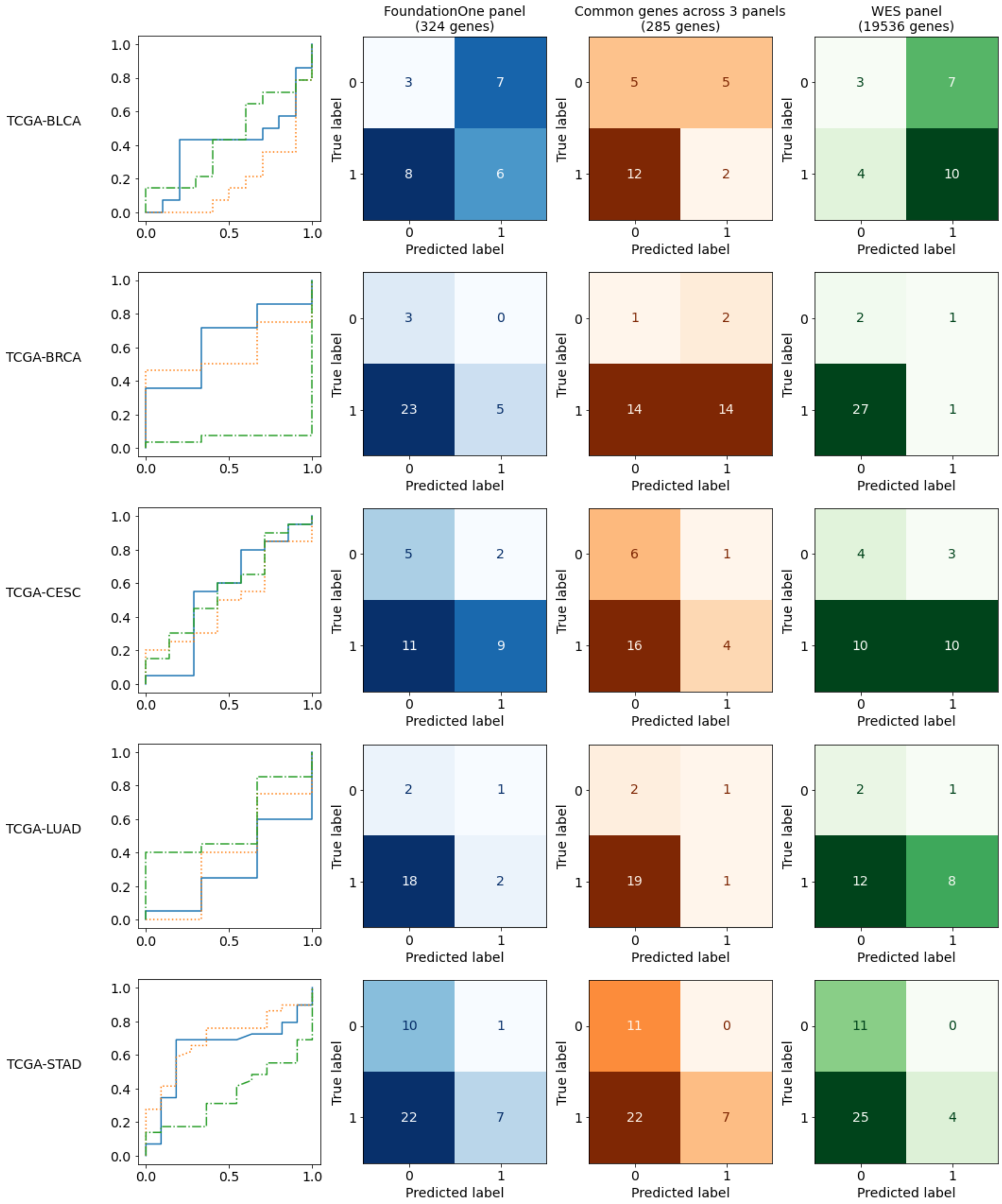

Figure S1: Comparison of CODE-AE performance across 3 cNGS panels distinguished by cancer type, related to STAR Methods (BLCA: Bladder Urothelial Carcinoma, BRCA: Breast invasive carcinoma, CESC: Cervical squamous cell carcinoma and endocervical adenocarcinoma, LUAD: Lung adenocarcinoma, STAD: Stomach adenocarcinoma, UCEC: Uterine Corpus Endometrial Carcinoma). Only cancer types with more than 20 (patient, drug) test pairs are considered here. In most cancer types, the confusion matrices look similar across all 3 cNGS panels suggesting a similar predictive performance of cNGS panels compared to WES.

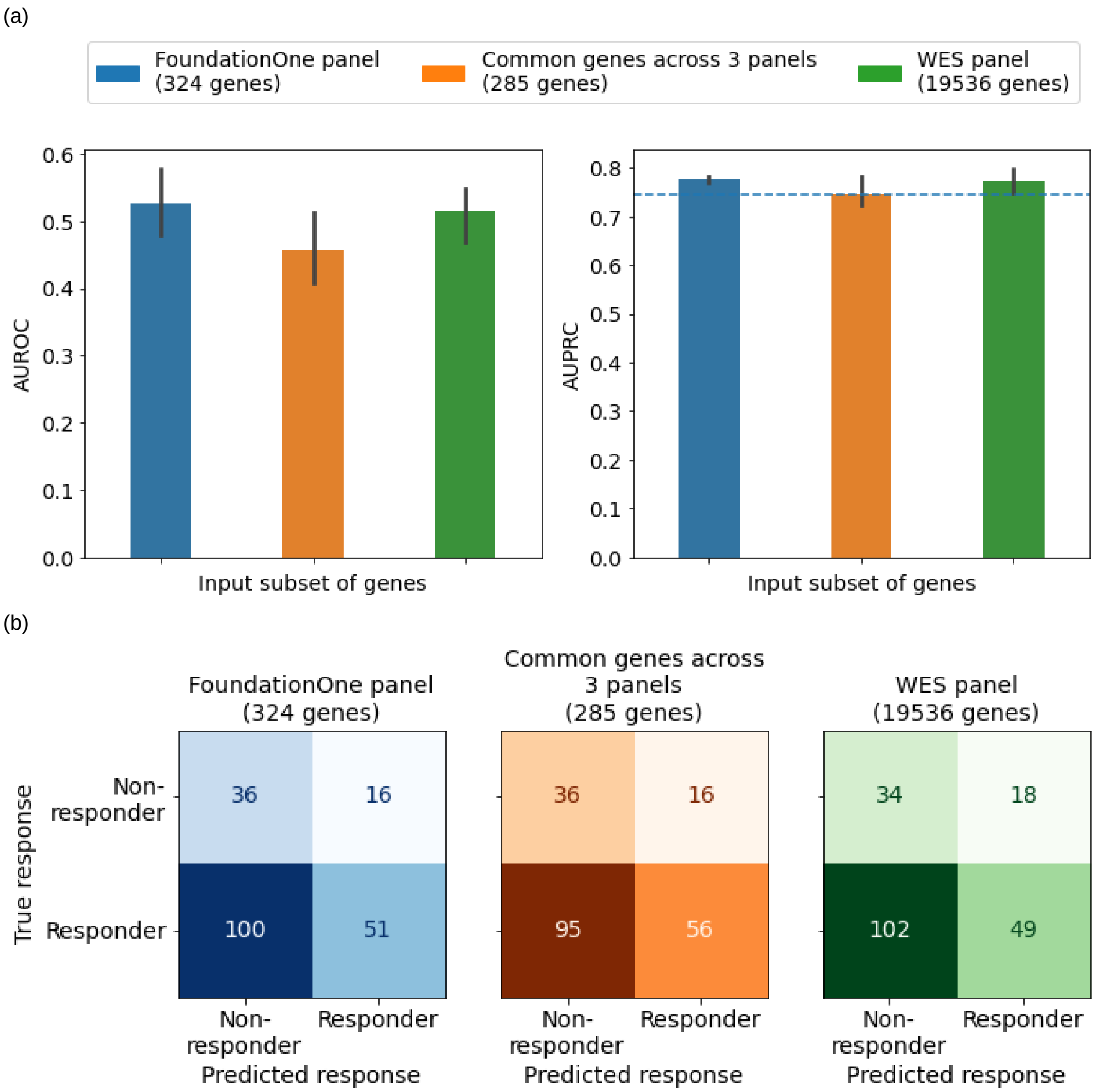

Figure S2: Comparison of performance for different input subsets of genes, related to STAR methods. (a) Comparison of AUROC and AUPRC scores of response prediction for different input subsets of genes. Performance is measured on 3 randomly chosen test splits, using TCGA data. Velodrome is used to predict response. Results show that performance is not significantly different (p-value associated with AUROC comparison: 0.259, p-value associated with AUPRC comparison: 0.281) across the 3 subsets of genes, suggesting that predictive value of a subset of genes used in cNGS panels is similar to that of all genes from WES. Baseline value for AUPRC: 0.7438. (b) Confusion matrices for different input subsets of genes on 203 samples from TCGA; predictions obtained using the method Velodrome. Predicted values were converted to binary responses using FPR and TPR thresholds of 0.3 each. Colour indicates the input subset, shade indicates magnitude of the values. All 3 subsets of genes yield similar distributions in the confusion matrices.

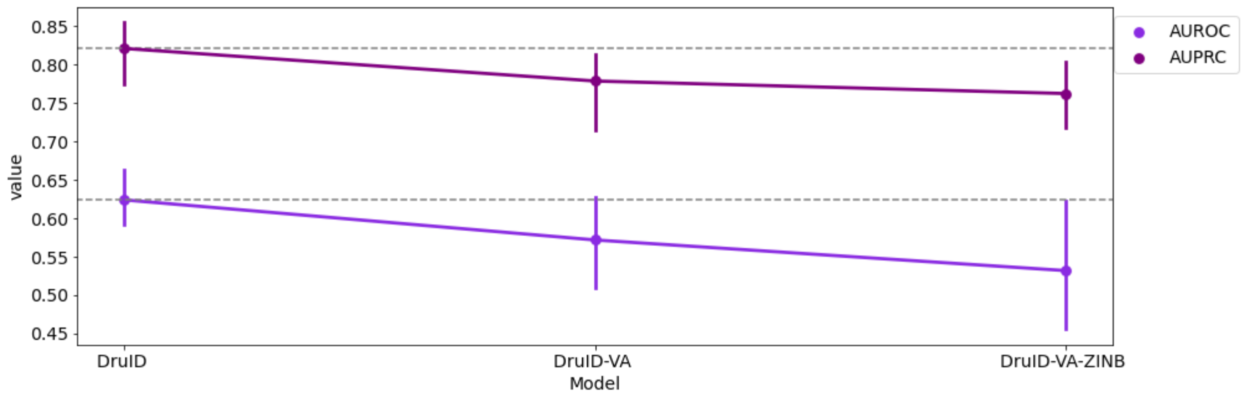

Figure S3: Ablation study with DruID, related to Results. Performance (AUPRC, above and AUROC, below) after incremental component-wise removal from DruID of variant annotation (DruID-VA) and zero inflated loss (DruID-VA-ZINB). Removal of each component reduces DruID's performance, thus showing the importance of each component.

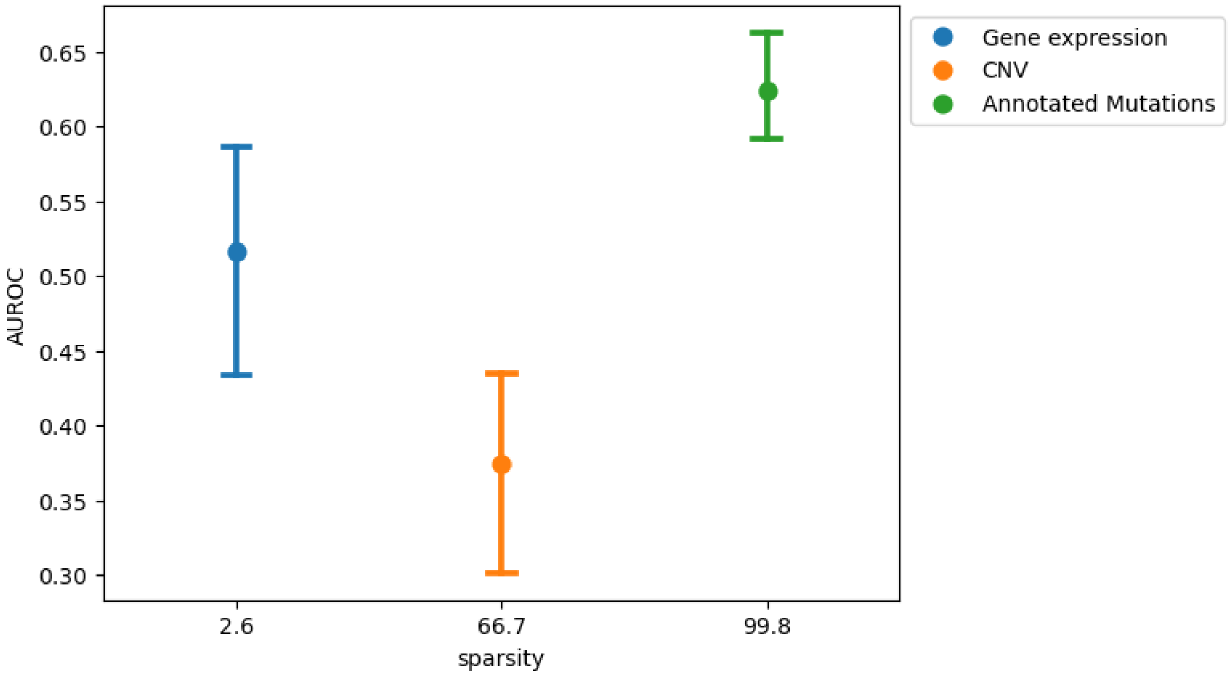

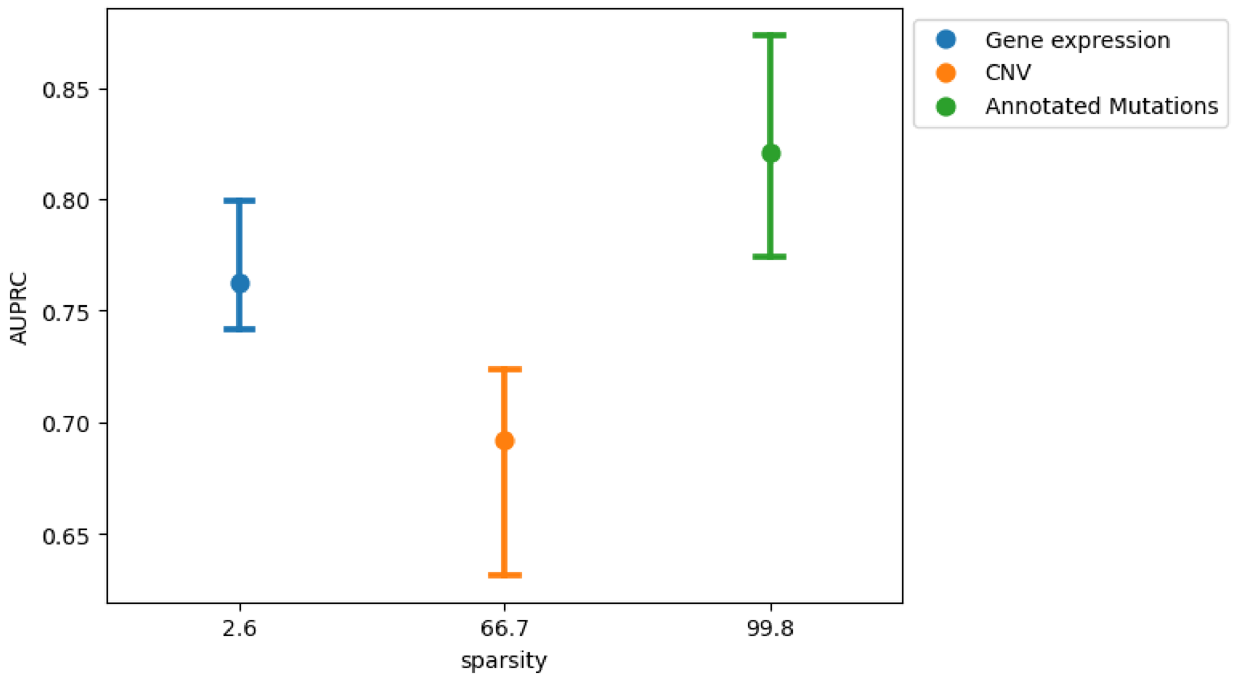

Figure S4: Comparison of performance, related to STAR methods (top: AUROC, bottom: AUPRC) across 3 test folds of TCGA patients, for various sparsity levels (rounded to 1st decimal place), ranging from 2.6% in gene expression, to 66.7% in copy number variations and 99.8% in annotated mutations.

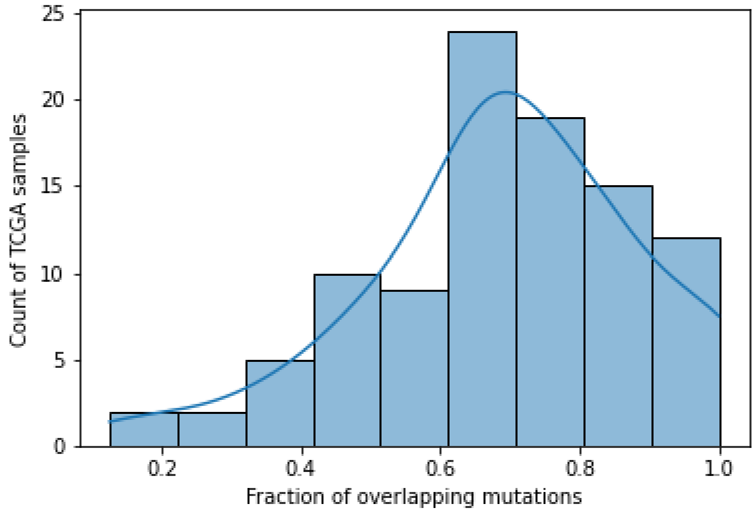

Figure S5: Plot indicates number of patients across various fractions of overlapping mutations, related to STAR methods. Fraction of overlapping mutations is the intersection over union of mutations present in the 324 FoundationOne CDx genes, after each variant calling approach. The left skew of the distribution and mean overlap of 69.16% indicate a substantial overlap in mutations identified across the two callers, for most patients. 71.43% samples have more than 60% overlap.

Table S1: Comparison of existing DRP methods and DruID, related to STAR Methods

Method	Variant Level Information Used	Use of unlabelled patient data in training	Evaluation on mutations	Modelling differences in drug response in patients and cell lines	Modelling distributional differences in omics inputs across patients and cell lines	Prediction possible on drugs not seen during training
CODE-AE[1] (He 2022)	✗	✓	✓	✗	✓	✗
Velodrome[2] (Sharifi 2021)	✗	✓	✗	✓	✓	✗
TUGDA[3] (Peres 2021)	✗	✓	✗	✗	✓	✓
TCRP[4] (Ma 2021)	✗	✗	✓	✗	✓	✗
AITL[5] (Sharifi 2020)	✗	✗	✗	✓	✓	✗
PRECISE[6] (Mourragui 2019)	✗	✓	✗	✗	✓	✗
TRANSACT[7] (Mourragui 2021)	✗	✓	✗	✗	✓	✗
PACE[8] (Anastopoulos 2021)	✗	✗	✗	✗	✓	✓
[9]Prasse 2022	✗	✗	✗	✗	✓	✓
[10]Tang 2022	✗	✗	✓	✓	✓	✓
DruID	✓	✓	✓	✓	✓	✓

Table compares and contrasts existing DRP methods with respect to whether they (1) use variant level information about mutations, (2) use mutations for training or evaluating their models, (3) utilise unlabelled patient data, (4) model differences in drug responses across patients and cell lines (5) handle distributional differences between patients and cell lines and (6) can predict on drugs that are not seen during training. While most methods handle distributional

differences between patients and cell lines, most do not handle the differences in drug response. Most methods do not use mutations and often do not utilise the available unlabelled data.

Table S2: Requirements and Design Choices in DruID, related to STAR methods

Design Requirements	Design Choices in DruID
Usage of Variant Level Information	Variant annotation procedure (Stage I) using existing bioinformatic tools like Annovar, GPD and ClinVar to encode the variant level information available in clinical sequencing reports achieves this.
Use of unlabelled patient data in training	DruID can utilise unlabelled patient data in the unsupervised domain-invariant representation learning phase (Stage II), using domain-specific VAEs.
Evaluation on mutations	Use of zero inflated distributions in VAE (Stage II) helps model sparsity in mutation data.
Modelling differences in drug response in patients and cell lines	Use of multi-task learning (Stage III) to model both AUDRC in cell lines and RECIST in patients.
Modelling distributional differences in omics inputs across patients and cell lines	Use of domain alignment CORAL loss to learn shared domain-invariant representations across both domains (Stage I)
Prediction possible on drugs not seen during training	Use of drug Morgan fingerprint as a model input allows inference on any new drug (Stage III), for which this input is available.

Table shows the shortcomings of prior methods, and the design choices made in the design of DruID's model architecture.

Table S3: CODE-AE performance across various subsets of genes, related to STAR methods.

	Foundation One (324 genes)	Common genes across 3 panels (285 genes)	WES panel (19,536 genes)
Sensitivity/Recall	0.272	0.291	0.311
Specificity	0.673	0.692	0.692
Precision	0.707	0.733	0.746

Sensitivity, Specificity and Precision values, from CODE-AE, corresponding to confusion matrices in Fig.1b.

Table S4: Velodrome performance across various subsets of genes, related to STAR methods.

	Foundation One (324 genes)	Common genes across 3 panels (285 genes)	WES panel (19,536 genes)
Sensitivity/Recall	0.3377	0.3709	0.3245
Specificity	0.6923	0.6923	0.6538
Precision	0.7612	0.7778	0.7313

Sensitivity, Specificity and Precision values, from Velodrome, corresponding to confusion matrices in Figure S2 (b).

Table S5: Mapping ClinVar annotations, related to STAR methods

Updated category	ClinVar generated annotations
Pathogenic	Pathogenic, Pathogenic|drug_response|other, Pathogenic/Likely_pathogenic, Likely_pathogenic, Pathogenic/Likely_pathogenic|other, drug_response, Likely_pathogenic|other, Pathogenic|risk_factor, Pathogenic/Likely_pathogenic|drug_response, Likely_risk_allele, risk_factor
Benign	Likely_benign, Benign/Likely_benign, Benign
Variants of Unknown Significance	., Uncertain_significance, Conflicting_interpretations_of_pathogenicity, not_provided, Conflicting_interpretations_of_pathogenicity|other, Uncertain_significance|drug_response, other

Mapping of clinical significance categories obtained from ClinVar to 3 broad annotation categories - pathogenic, benign and variants of unknown significance.

Table S6: Description of AnnoVar annotations, related to STAR methods

Algorithm	Description
SIFT[11]	SIFT (Sorting Intolerant From Tolerant) uses sequence homology and the physical properties of amino acids to predict whether an amino acid substitution affects protein function; D: Deleterious (sift<=0.05); T: tolerated (sift>0.05)
SIFT4G[11]	SIFT 4G is a faster version of SIFT that scales up and provides SIFT predictions for more organisms
LRT[12]	LRT (Likelihood Ratio Test) uses comparative genomics to identify variants that disrupt highly conserved amino acids within protein-coding sequences; D: Deleterious; N: Neutral; U:

	Unknown
MutationTaster[13]	MutationTaster applies a naive Bayes classifier eventually predict the disease potential of an alteration; "A" ("disease_causing_automatic"); "D" ("disease_causing"); "N" ("polymorphism"); "P" ("polymorphism_automatic"); A, D signifies deleterious alterations
MutationAssessor[14]	MutationAssessor is based on evolutionary conservation of the affected amino acid in protein homologs by combinatorial entropy formalism to compute a Functional Impact Score (FIS); H: high; M: medium; L: low; N: neutral. H/M signifies functional alterations and L/N signifies non-functional alterations
FATHMM[15]	Predicts the functional, molecular, and phenotypic consequences of amino acid substitutions using hidden Markov models; D: Deleterious (<=-1.5); T: tolerated (>1.5)
PROVEAN[16]	PROVEAN (Protein Variation Effect Analyzer) provides a generalized approach to predict the functional effects of protein sequence variations including single or multiple amino acid substitutions, and in-frame insertions and deletions; D: Deleterious (<=-2.282); N: neutral(>-2.282)
MetaSVM[17]	Similar to SIFT but less missing values; D: Deleterious; T: Tolerated
M-CAP[18]	M-CAP is a classifier for rare missense variants in the human genome that is tuned to the high sensitivity required in the clinic (combines previous pathogenicity scores (including SIFT, Polyphen-2 and CADD) with novel features and a powerful model); D: Deleterious (>=0.025); T: tolerated (<0.025)
PrimateAI[19]	Deep residual neural network for classifying the pathogenicity of missense mutations; D: Deleterious (>=0.803); T: tolerated (<0.803)
DEOGEN2[20]	DEOGEN2 incorporates heterogeneous information about the molecular effects of the variants, the domains involved, the relevance of the gene and the interactions in which it participates. This is then non-linearly mapped into one single deleteriousness score for each variant; D: Deleterious (>=0.45); T: tolerated (<0.45)
BayesDel - AF[21]	BayesDel is a deleteriousness meta-score. It works for coding

	and non-coding variants, single nucleotide variants and small insertion / deletions. The range of the score is from -1.29334 to 0.75731. The higher the score, the more likely the variant is pathogenic; For MaxAF -> D: Deleterious (>=0.0692); T: tolerated (<0.0692)
BayesDel - noAF[21]	BayesDel is a deleteriousness meta-score. It works for coding and non-coding variants, single nucleotide variants and small insertion / deletions. The range of the score is from -1.29334 to 0.75731. The higher the score, the more likely the variant is pathogenic; Without MaxAF -> D: Deleterious (>=-0.0570); T: tolerated (<-0.0570)
ClinPred[22]	Prediction Tool to Identify Disease-Relevant Nonsynonymous Single-Nucleotide Variants; D: Deleterious (>=0.5); T: tolerated (<0.5)
LIST-S2[23]	Taxonomy based sorting of deleterious missense mutations across species where e higher scores imply higher potential deleteriousness; D: Deleterious (>=0.85); T: tolerated (<0.85)
FATHMM MKL[24]	Predicts the functional consequences of both coding and non-coding sequence variants utilizing various genomic annotations, which have recently become available, and learns to weight the significance of each component annotation source; D: Deleterious (>=0.5); T: tolerated (<0.5)
FATHMM XF[25]	FATHMM with an eXtended Feature set (FATHMM-XF) which yields highly accurate predictions for SNVs across the entire human genome; D: Deleterious (>=0.5); T: tolerated (<0.5)

Description of the 17 prediction algorithms used by Annovar to annotate a given mutation. These algorithms broadly flag mutations as deleterious or tolerated, which is further encoded as a binary outcome as part of DruID processing in Stage I variant annotations.

Table S7: CCLE-TCGA train-test split 0 statistics, related to STAR methods

Drug Name	Train split TCGA	Test split TCGA	Train split CCLE	Test split CCLE
CISPLATIN	167	39	425	112
PACLITAXEL	87	26	542	134
5-FLUOROURACIL	100	25	468	121

OVERALL	354	90	1435	367

Number of (patient, drug) pairs in the train and test splits of CCLE-TCGA dataset split 0. Each cell indicates the number of patients/samples who were treated with the corresponding drug, which are further divided up across the train and test splits.

Table S8: Overall - cancer type distribution in CCLE-TCGA train-test split 0, related to STAR methods

Cancer type	Train split TCGA	Test split TCGA
BLCA	48	8
BRCA	56	21
CESC	49	13
COAD	27	7
HNSC	46	7
LUAD	39	12
LUSC	22	3
SKCM	10	1
STAD	59	16
UCEC	27	7

Number of (patient, drug) pairs in the train and test splits of TCGA dataset split 0. Each cell indicates the number of patients belonging to the specific cancer type indicated by the row, which are further divided up across the train and test splits.

Table S9: Drug specific - cancer type distribution (TCGA), related to STAR methods

Drug	Cancer type	Train split TCGA	Test split TCGA
CISPLATIN	BLCA	32	4
	CESC	39	11
	HNSC	32	6
	LUAD	27	10
	LUSC	14	1
	SKCM	7	0

	STAD	12	7
	UCEC	4	0
PACLITAXEL	BLCA	8	0
	BRCA	19	12
	CESC	6	2
	HNSC	12	1
	LUAD	11	2
	LUSC	2	1
	SKCM	1	1
	STAD	3	0
	UCEC	25	7
5-FLUOROURACIL	BLCA	1	0
	BRCA	16	5
	CESC	4	0
	COAD	27	7
	HNSC	1	0
	STAD	51	13

Number of (patient, drug) pairs in the train and test splits of TCGA dataset split 0. Each cell indicates the number of patients belonging to the specific cancer type indicated by the row, which are further divided up across the train and test splits. These are also categorised based on the drug administered in each case.

Table S10: CCLE-CRC train-test split 0 statistics, related to STAR methods

Drug Name	Train split NUH CRC	Test split NUH CRC	Train split CCLE	Test split CCLE
5-FLUOROURACIL	65	17	468	121
IRINOTECAN	20	10	531	137
OXALIPLATIN	44	7	441	114
OVERALL	129	34	1440	372

Number of (patient, drug) pairs in the train and test splits of CCLE-NUH CRC dataset split 0. Each cell indicates the number of patients/samples who were treated with the corresponding drug, which are further divided up across the train and test splits.

Table S11: CCLE-OV train-test split 0 statistics, related to STAR methods

Drug Name	Train split NUH Ovarian	Test split NUH Ovarian	Train split CCLE	Test split CCLE
CISPLATIN	88	17	425	112
PACLITAXEL	87	15	540	136
GEMCITABINE			425	112
DOXORUBICIN			447	110
OVERALL	175	32	1837	470

Number of (patient, drug) pairs in the train and test splits of CCLE-NUH ovarian dataset split 0. Each cell indicates the number of patients/samples who were treated with the corresponding drug, which are further divided up across the train and test splits.

Table S12: CCLE-TCGA train-test split 1 statistics, related to STAR methods

Drug Name	Train split TCGA	Test split TCGA	Train split CCLE	Test split CCLE
CISPLATIN	168	38	426	111
PACLITAXEL	87	26	538	138
5-FLUOROURACIL	107	18	472	117
OVERALL	362	82	1436	366

Number of (patient, drug) pairs in the train and test splits of CCLE-TCGA dataset split 1. Each cell indicates the number of patients/samples who were treated with the corresponding drug, which are further divided up across the train and test splits.

Table S13: CCLE-CRC train-test split 1 statistics, related to STAR methods

Drug Name	Train split NUH CRC	Test split NUH CRC	Train split CCLE	Test split CCLE
5-FLUOROURACIL	65	17	468	121

IRINOTECAN	22	8	532	136
OXALIPLATIN	43	8	440	115
OVERALL	130	33	1440	372

Number of (patient, drug) pairs in the train and test splits of CCLE-NUH CRC dataset split 1. Each cell indicates the number of patients/samples who were treated with the corresponding drug, which are further divided up across the train and test splits.

Table S14: CCLE-OV train-test split 1 statistics, related to STAR methods

Drug Name	Train split NUH Ovarian	Test split NUH Ovarian	Train split CCLE	Test split CCLE
CISPLATIN	73	32	430	107
PACLITAXEL	70	32	540	136
GEMCITABINE	1		430	107
DOXORUBICIN			449	108
OVERALL	144	64	1849	458

Number of (patient, drug) pairs in the train and test splits of CCLE-NUH Ovarian dataset split 1. Each cell indicates the number of patients/samples who were treated with the corresponding drug, which are further divided up across the train and test splits.

Table S15: CCLE-TCGA train-test split 2 statistics, related to STAR methods

Drug Name	Train split TCGA	Test split TCGA	Train split CCLE	Test split CCLE
CISPLATIN	167	39	431	106
PACLITAXEL	86	27	541	135
5-FLUOROURACIL	101	24	473	116
OVERALL	354	90	1445	357

Number of (patient, drug) pairs in the train and test splits of CCLE-TCGA dataset split 2. Each cell indicates the number of patients/samples who were treated with the corresponding drug, which are further divided up across the train and test splits.

Table S16: CCLE-CRC train-test split 2 statistics, related to STAR methods

Drug Name	Train split NUH CRC	Test split NUH CRC	Train split CCLE	Test split CCLE
5-FLUOROURACIL	65	17	473	116
IRINOTECAN	24	6	534	134
OXALIPLATIN	39	12	451	104
OVERALL	128	35	1458	354

Number of (patient, drug) pairs in the train and test splits of CCLE-NUH CRC dataset split 2. Each cell indicates the number of patients/samples who were treated with the corresponding drug, which are further divided up across the train and test splits.

Table S17: CCLE-OV train-test split 2 statistics, related to STAR methods

Drug Name	Train split NUH Ovarian	Test split NUH Ovarian	Train split CCLE	Test split CCLE
CISPLATIN	89	16	430	107
PACLITAXEL	86	16	540	136
GEMCITABINE	1		430	107
DOXORUBICIN			449	108
OVERALL	176	32	1849	458

Number of (patient, drug) pairs in the train and test splits of CCLE-NUH Ovarian dataset split 2. Each cell indicates the number of patients/samples who were treated with the corresponding drug, which are further divided up across the train and test splits.

Table S18: Sparsity statistics, related to STAR methods

Dataset name	Data type (TCGA)	Number of train samples (N) (sample, drug pairs)	Number of features (F)	Sparsity (%)
TCGA	Annotated mutations	503	7776	99.8208
	Gene expression	364	324	2.5819
	Copy number variation	503	972	66.6667

IMAC-OV	Annotated mutations	175	7776	99.353
IMAC-CRC	Annotated mutations	140	7776	98.9569

Sparsity in train data for various input data types, across TCGA, IMAC-OV and IMAC-CRC (split 0). 'Number of samples' includes all the train samples, with corresponding features in the datasets used in all experiments.

Table S19: Summary of input features used, related to STAR methods

Input Type	Feature Set	Encoding
Mutations (F1 genes) vector	324 genes sequenced in FoundationOne report	324-dimensional binary vector with 1 bit per gene sequenced. A value of 1 indicates presence of mutation in a gene and 0 indicates its absence.
Mutations (All genes) vector	All 19536 genes sequenced	19536-dimensional binary vector with 1 bit per gene sequenced. A value of 1 indicates presence of mutation in a gene and 0 indicates its absence.
Mutations (285 genes) vector	285 genes sequenced in FoundationOne, Tempus xF+ and TruSight Oncology 500 reports	285-dimensional binary vector with 1 bit per gene sequenced. A value of 1 indicates presence of mutation in a gene and 0 indicates its absence.
Gene expression (F1 genes) vector	324 genes sequenced in FoundationOne report	324-dimensional real-valued vector with 1 dimension per gene sequenced. No additional encoding done over raw data.
Copy number variation CNV (F1 genes) vector	324 genes sequenced in FoundationOne report	972-dimensional binary vector with 3 bits per gene sequenced. Raw data encoded with -1 indicating loss, +1 indicating amplification and 0 indicating no change. This was further one hot encoded to obtain 3 bits per gene.
Combined CNV and annotated mutation (F1 genes) vector	324 genes sequenced in FoundationOne report	Obtained by concatenating VAE encoded representation for mutations (F1 genes) vector and VAE encoded representation for copy number variation CNV (F1 genes) vector.
Combined gene expression and annotated mutation (F1 genes) vector	324 genes sequenced in FoundationOne report	8100-dimensional vector obtained by concatenating annotated mutations (F1 genes) vector and gene expression (F1 genes) vector.
Variant annotated mutation (F1 genes)	324 genes sequenced in FoundationOne report	7776-dimensional vector obtained after variant annotation using Annovar, GPD

vector		and ClinVar, followed by an aggregation across all mutations in each gene.

Summary of various input data types used across all the experiments in this paper. Mutations, copy number variations, gene expression and annotated mutation vectors are the key input data types. We further create different subsets based on the number of genes considered in each case. The resulting dimensions of the feature vectors are also described, in each case.

Table S20: Key hyperparameters used to train DruID, related to STAR Methods

Stage II Unsupervised Domain Invariant Representation Learning			
Hyperparameter	CCLE-TCGA	CCLE-CRC	CCLE-OV
VAE hidden layer dimensions (encoder and decoder)	No pre-training; uses model pre-trained on CCLE-IMACGO for fine tuning	[128, 64]	[128, 64]
VAE activation function		tanh	tanh
Learning Rate		1e-5	1e-5
Epochs		1000	1000
Convergence threshold		1e-5	1e-5
Stage III Multi-task Drug Response Prediction			
Hyperparameter	CCLE-TCGA (fine-tuning)	CCLE-CRC	CCLE-OV
Batch size	256	256	256
Epochs	50	500	500
Cell line embedder learning rate	1e-4	1e-4	1e-6
Patient embedder learning rate	1e-4 for 5-fu; 1e-3 for Cisplatin and Paclitaxel	1e-3	1e-4
AUDRC and RECIST predictor learning rate	1e-3 for 5-fu; 1e-5 for Cisplatin; 1e-6 for Paclitaxel	1e-6	1e-6

Drug embedder learning rate	1e-4	1e-4	1e-4

Table shows the key hyperparameters used to train DruID, specifically in stages II and III on train split 0 for each dataset used in the experiments. For CCLE-TCGA dataset, the initial pre-training is done on the CCLE and IMAC-GO datasets with further fine-tuning done per drug. In all other cases, initial pre-training and fine-tuning are done on the same dataset.

Table S21: Comparison across DRP methods on gene expression data, related to STAR methods

AUROC scores					
Drug	TCRP	TUGDA	Velodrome	CODE-AE	DruID
SORAFENIB	0.5482 +- 0.3445	0.4786 +- 0.3203	0.5482 +- 0.3066	0.3704 +- 0.3208	**0.6889 +- 0.3006**
CISPLATIN	0.6222 +- 0.4018	0.512 +- 0.2038	0.2984 +- 0.1507	0.4127 +- 0.1915	**0.8222 +- 0.1678**
GEMCITABINE	0.5347 +- 0.1185	0.432 +- 0.0944	0.4216 +- 0.1713	0.4474 +- 0.2252	**0.6984 +- 0.2374**
TEMOZOLOMIDE	0.6984 +- 0.0999	0.4716 +- 0.1375	0.7401 +- 0.0653	**0.9127 +- 0.0422**	0.6548 +- 0.2407
5-FLUOROURACIL	0.7222 +- 0.347	0.3684 +- 0.1255	0.3889 +- 0.2546	0.6111 +- 0.2546	**0.7778 +- 0.0962**
AUPRC scores					
Drug	TCRP	TUGDA	Velodrome	CODE-AE	DruID
SORAFENIB	0.613 +- 0.3706	0.086 +- 0.0258	0.6333 +- 0.3756	0.537 +- 0.3207	**0.6944 +- 0.3938**
CISPLATIN	0.6909 +- 0.2677	0.0984 +- 0.0144	0.3555 +- 0.1869	0.4219 +- 0.2401	**0.9056 +- 0.1055**
GEMCITABINE	0.7391 +- 0.2081	0.2104 +- 0.1009	0.65 +- 0.2173	0.6551 +- 0.1409	**0.8119 +- 0.1544**
TEMOZOLOMIDE	0.7714 +- 0.1482	0.204 +- 0.1348	0.7579 +- 0.1835	**0.9222 +- 0.0592**	0.7818 +- 0.0589
5-FLUOROURACIL	0.7611 +- 0.282	0.0609 +- 0.012	0.4556 +- 0.1134	0.6111 +- 0.2097	**0.8055 +- 0.0481**

AUROC and AUPRC scores (mean+- SD) across 5 drugs, over 3 test folds. DruID outperforms baselines in 4 out of drugs in both metrics.

Table S22: DruID Performance on drugs not seen during training (TCGA), related to STAR methods

Drug Name	R/NR	AUROC	AUPRC	F1	Accuracy	Precision	Recall	Baseline AUPRC (P/N)
Doxorubicin	22/5	0.6182	0.833	0.8889	0.8148	0.8696	0.9091	0.8148
Vinorelbine	15/1	1	1	0.9655	0.9375	1	0.9333	0.9375

Table shows AUROC and AUPRC performance metrics for (patient, drug) pairs where the drug does not appear in the train data. R/NR refers to the number of responders and non-responders in the inference dataset. Baseline AUPRC refers to the ratio of positive samples to the total number of samples in the inference dataset.

Table S23: Comparison of DruID when trained with all cancer types, related to STAR methods

**Drug Name**	**With 10 cancer types (With filtration step)**	**With all cancer types (Without filtration step)**
AUROC		
Cisplatin	0.6738 +- 0.1063	0.3985 +- 0.1042
Paclitaxel	0.6343 +- 0.0497	0.4907 +- 0.1104
5-Fluorouracil	0.6473 +- 0.0872	0.6739 +- 0.1036
AUPRC		
Cisplatin	0.863 +- 0.0735	0.7574 +- 0.047
Paclitaxel	0.8255 +- 0.0683	0.7454 +- 0.0326
5-Fluorouracil	0.8555 +- 0.0643	0.8666 +- 0.0687

Table shows the performance in AUROC and AUPRC across 3 test folds, when DruID is trained with only 10 cancer types and when trained with all cancer types.

References

1. He, D., and Xie, L. (2021). CODE-AE: A Coherent De-confounding Autoencoder for Predicting Patient-Specific Drug Response From Cell Line Transcriptomics. Preprint at arXiv.

2. Sharifi-Noghabi, H., Harjandi, P.A., Zolotareva, O., Collins, C.C., and Ester, M. (2021). Out-of-distribution generalization from labelled and unlabelled gene expression data for drug response prediction. Nat. Mach. Intell. *3*, 962–972. https://doi.org/10.1038/s42256-021-00408-w.

3. Peres da Silva, R., Suphavilai, C., and Nagarajan, N. (2021). TUGDA: task uncertainty guided domain adaptation for robust generalization of cancer drug response prediction from *in vitro* to *in vivo* settings. Bioinformatics *37*, i76–i83. https://doi.org/10.1093/bioinformatics/btab299.

4. Ma, J., Fong, S.H., Luo, Y., Bakkenist, C.J., Shen, J.P., Mourragui, S., Wessels, L.F.A., Hafner, M., Sharan, R., Peng, J., et al. (2021). Few-shot learning creates predictive models of drug response that translate from high-throughput screens to individual patients. Nat. Cancer *2*, 233–244. https://doi.org/10.1038/s43018-020-00169-2.

5. AITL: Adversarial Inductive Transfer Learning with input and output space adaptation for pharmacogenomics.

6. Mourragui, S., Loog, M., van de Wiel, M.A., Reinders, M.J.T., and Wessels, L.F.A. (2019). PRECISE: a domain adaptation approach to transfer predictors of drug response from pre-clinical models to tumors. Bioinformatics *35*, i510–i519. https://doi.org/10.1093/bioinformatics/btz372.

7. Mourragui, S., and Loog, M. (2021). TRANSACT. PNAS.

8. Anastopoulos, I., Seninge, L., Ding, H., and Stuart, J. (2021). Patient Informed Domain Adaptation Improves Clinical Drug Response Prediction (Bioinformatics) https://doi.org/10.1101/2021.08.07.455527.

9. Prasse, P., Iversen, P., Lienhard, M., Thedinga, K., Herwig, R., and Scheffer, T. (2022). Pre-Training on In Vitro and Fine-Tuning on Patient-Derived Data Improves Deep Neural Networks for Anti-Cancer Drug-Sensitivity Prediction. Cancers *14*, 3950. https://doi.org/10.3390/cancers14163950.

10. Tang, Y.-C., and Gottlieb, A. (2021). Explainable drug sensitivity prediction through cancer pathway enrichment. Sci. Rep. *11*, 3128. https://doi.org/10.1038/s41598-021-82612-7.

11. SIFT - Predict effects of nonsynonmous / missense variants https://sift.bii.a-star.edu.sg/.

12. Chun, S., and Fay, J.C. (2009). Identification of deleterious mutations within three human genomes. Genome Res. *19*, 1553–1561. https://doi.org/10.1101/gr.092619.109.

13. Schwarz, J.M., Rödelsperger, C., Schuelke, M., and Seelow, D. (2010). MutationTaster evaluates disease-causing potential of sequence alterations. Nat. Methods *7*, 575–576. https://doi.org/10.1038/nmeth0810-575.

14. Reva, B., Antipin, Y., and Sander, C. (2011). Predicting the functional impact of protein mutations: application to cancer genomics. Nucleic Acids Res. *39*, e118. https://doi.org/10.1093/nar/gkr407.

15. Shihab, H.A., Gough, J., Cooper, D.N., Stenson, P.D., Barker, G.L.A., Edwards, K.J., Day, I.N.M., and Gaunt, T.R. (2013). Predicting the functional, molecular, and phenotypic consequences of amino acid substitutions using hidden Markov models. Hum. Mutat. *34*, 57–65. https://doi.org/10.1002/humu.22225.

16. Choi, Y., and Chan, A.P. (2015). PROVEAN web server: a tool to predict the functional effect of amino acid substitutions and indels. Bioinforma. Oxf. Engl. *31*, 2745–2747. https://doi.org/10.1093/bioinformatics/btv195.

17. Dong, C., Wei, P., Jian, X., Gibbs, R., Boerwinkle, E., Wang, K., and Liu, X. (2015). Comparison and integration of deleteriousness prediction methods for nonsynonymous SNVs in whole exome sequencing studies. Hum. Mol. Genet. *24*, 2125–2137. https://doi.org/10.1093/hmg/ddu733.

18. Jagadeesh, K.A., Wenger, A.M., Berger, M.J., Guturu, H., Stenson, P.D., Cooper, D.N., Bernstein, J.A., and Bejerano, G. (2016). M-CAP eliminates a majority of variants of uncertain significance in clinical exomes at high sensitivity. Nat. Genet. *48*, 1581–1586. https://doi.org/10.1038/ng.3703.

19. Sundaram, L., Gao, H., Padigepati, S.R., McRae, J.F., Li, Y., Kosmicki, J.A., Fritzilas, N., Hakenberg, J., Dutta, A., Shon, J., et al. (2018). Predicting the clinical impact of human mutation with deep neural networks. Nat. Genet. *50*, 1161–1170. https://doi.org/10.1038/s41588-018-0167-z.

20. Raimondi, D., Tanyalcin, I., Ferté, J., Gazzo, A., Orlando, G., Lenaerts, T., Rooman, M., and Vranken, W. (2017). DEOGEN2: prediction and interactive visualization of single amino acid variant deleteriousness in human proteins. Nucleic Acids Res. *45*, W201–W206. https://doi.org/10.1093/nar/gkx390.

21. Tian, Y., Pesaran, T., Chamberlin, A., Fenwick, R.B., Li, S., Gau, C.-L., Chao, E.C., Lu, H.-M., Black, M.H., and Qian, D. (2019). REVEL and BayesDel outperform other in silico meta-predictors for clinical variant classification. Sci. Rep. *9*, 12752. https://doi.org/10.1038/s41598-019-49224-8.

22. Alirezaie, N., Kernohan, K.D., Hartley, T., Majewski, J., and Hocking, T.D. (2018). ClinPred: Prediction Tool to Identify Disease-Relevant Nonsynonymous Single-Nucleotide Variants. Am. J. Hum. Genet. *103*, 474–483. https://doi.org/10.1016/j.ajhg.2018.08.005.

23. Malhis, N., Jacobson, M., Jones, S.J.M., and Gsponer, J. (2020). LIST-S2: taxonomy based sorting of deleterious missense mutations across species. Nucleic Acids Res. *48*, W154–W161. https://doi.org/10.1093/nar/gkaa288.

24. Shihab, H.A., Rogers, M.F., Gough, J., Mort, M., Cooper, D.N., Day, I.N.M., Gaunt, T.R., and Campbell, C. (2015). An integrative approach to predicting the functional effects of non-coding and coding sequence variation. Bioinforma. Oxf. Engl. *31*, 1536–1543. https://doi.org/10.1093/bioinformatics/btv009.

25. Rogers, M.F., Shihab, H.A., Mort, M., Cooper, D.N., Gaunt, T.R., and Campbell, C. (2018). FATHMM-XF: accurate prediction of pathogenic point mutations via extended features. Bioinforma. Oxf. Engl. *34*, 511–513. https://doi.org/10.1093/bioinformatics/btx536.
